# Association of Sleep Duration and Weekend Catch-Up Sleep with Suicidal Ideation among Adolescents with Atopic Dermatitis

**DOI:** 10.3390/jcm12247716

**Published:** 2023-12-15

**Authors:** Dong Wan Kang, Sung Hoon Kim, Yechan Kyung, Hae Jeong Lee

**Affiliations:** Department of Pediatrics, Samsung Changwon Hospital, Sungkyunkwan University School of Medicine, Changwon 51353, Republic of Koreahee7307@hanmail.net (S.H.K.);

**Keywords:** atopic dermatitis, sleep, weekend catch-up sleep, suicidal ideation

## Abstract

Atopic dermatitis (AD) is a prevalent allergic disease in children that often causes sleep disturbance and which is associated with diminished quality of life and heightened susceptibility to depression and suicidality. In this study, we investigate the relationship between weekend catch-up sleep (WCUS) and mental health in a sample of 71,434 adolescents with AD and 221,057 without AD using multivariate logistic regression analysis. We hypothesized that adolescents with AD experience shorter total and daytime sleep due to pruritus, with compensatory longer WCUS. We found that the lifetime prevalence of AD was 24.4%, and that adolescents with AD slept less overall, with significantly shorter weekday and weekend sleep durations but longer weekend catch-up sleep (WCUS). Sleep dissatisfaction was markedly higher in the AD group. Interestingly, our results suggest that prolonged WCUS is a protective factor against suicidal ideation in adolescents with AD. The study highlights the importance of addressing sleep patterns in adolescents with AD to enhance mental health. Overall, our findings indicate a need for increased awareness and intervention strategies to ensure sufficient sleep and reduce the risk of suicidal ideation in this population.

## 1. Introduction

Atopic dermatitis (AD) is a common allergic disease with an estimated prevalence of up to 20% in children and up to 3% in adults [1]. It is a chronically recurrent pruritic inflammatory skin disease with complex pathophysiology that is still not fully understood [2,3]. AD causes itching and scratching, pain, bleeding, and secondary bacterial infection [4,5,6], and impairs quality of life in a number of ways, one of which is by impacting sleep [2,7]. Itching is a problem for approximately 60–90% of adults with AD, and is often characterized as the most burdensome symptom of the disease [7].

Sleep disturbance is very common, reported for 47% to 80% of children and 33% to 87.1% of adults with AD [2,7]. According to a prior meta-analysis [7], itching and pain can impair sleep quality and result in the most commonly reported sleep problems in both children and adults with AD, namely, trouble falling asleep (10.2% to 51.4% in children, 80% in adults), frequent nighttime awakenings (43% to 73% in children, 4.8% with mild disease and 92.6% with severe disease in adults), and excessive daytime sleepiness (43.1% to 61.9% in children) [4,5,6]. Sleep disturbance is a major factor leading to impaired quality of life, including depression and suicidality, and is often viewed as a measure of disease severity in AD [7,8].

Weekend catch-up sleep (WCUS) is defined as sleep time on weekends that exceeds sleep time on weekdays and is an indicator of insufficient weekday sleep [9]. Adolescents not only tend to have shorter sleep duration during the night compared with children, but also tend to sleep less during school days due to the pressure of schoolwork, while sleeping more on weekends to compensate for insufficient weekday sleep during the entire week [10,11,12]. However, the effects of WCUS on health are conflicting: WCUS has been associated with lowered risk of hypertension [13], obesity prevention [14], and with beneficial effects for low-grade systemic inflammation in adults [3]. However, it has also been shown not to ameliorate the risk of obesity [15], to have adverse effects on executive functions [16], and be associated with poor performance on objective attention tasks [17] as well as with suicide attempts and self-injury in children and adolescents [9]. The consequences of AD are not limited to the appearance and feel of the skin [18,19,20]. AD impacts quality of life, including social, psychological, academic, economic, and occupational aspects [18]. Chronic dermatologic illnesses, including AD, can be associated with high levels of anxiety, depressive symptoms, and suicidal ideation [21,22]. Previous studies have found that patients with AD are 44% more likely to have suicidal ideation and 36% more likely to attempt suicide in comparison with patients without AD [23,24]. Recent years have seen a rapid increase in suicide rates in adolescents, causing suicide to be among the five most common causes of adolescent death worldwide [25]. In South Korea, despite the implementation of a national suicide prevention program in the early 2000s, suicide has been the most common cause (30.9%) of death in Korean adolescents since 2016 [26,27].

Suicide is a prevalent cause of death during adolescence, and there is a well-established connection between insufficient sleep and suicidal tendencies. Meta-analyses by Liu et al. revealed significantly increased risks of suicidal ideation, plans, and attempts in adolescents with sleep disturbances [28]. Another meta-analysis showed that shorter sleep durations in adolescents are associated with higher rates of suicidal ideation and attempts [29]. However, to date there have been no studies on the relationship between WCUS and AD in adolescents or on the question of whether WCUS influences suicidal ideation in adolescents with AD. Because current suicidal ideation is the most important predictor of future suicide attempts [30], and adolescents with AD have a higher prevalence of suicidal behaviors [31], identifying sleep factors associated with suicidal ideation in adolescents with AD may assist with the effort to reduce suicide in this group. It is well documented that factors like skipping breakfast, lack of exercise, and smoking can influence the mental health status of individuals, including those with AD [32]. Therefore, in this study we explore the potential associations between these additional factors and suicidal ideation in adolescents with AD, given their known impact on the mental health of individuals.

We hypothesized that adolescents with AD would experience shorter total and daytime sleep duration due to itching, while compensating with longer weekend sleep duration and a more extended WCUS to make up for insufficient sleep during the week, compared with adolescents without AD. We had two main goals in this study. First, we conducted a comparative analysis examining differences in clinical characteristics, sleep patterns, and mental health status between adolescents with and without AD. Second, our primary focus was identifying sleep factors associated with suicidal ideation in adolescents with AD.

## 2. Materials and Methods

### 2.1. Study Design and Participants

This study was designed as a secondary data analysis of data drawn from the third through eleventh Korean Youth Risk Behavior Web-based Surveys (KYRBSs) conducted during a five year period from 2013 to 2017. Initiated in 2005 by the Korean Ministry of Education, Ministry of Health and Welfare, and Centers for Disease Control and Prevention (KCDC), the KYRBS is an annual a cross-sectional, self-administered, anonymous online survey, encompassing a nationally representative sample of Korean adolescents aged 12–18 years. In our study, we included the final sample that comprised 71,434 adolescents with atopic dermatitis (AD) and 221,057 adolescents without AD. The mean age of the participants was 15.01 ± 1.75 years. The Institutional Review Board (IRB) of the KCDC approved this online statistical research for the entire duration of the survey.

The KYRBS utilizes the stratified three stage random cluster sampling method. The study population underwent stratification based on geographic region and school type in the first stage to minimize sampling errors. In the second stage, 400 middle schools and 400 high schools were selected through proportional sampling to match the study population every year. Finally, in the third stage, sample classes were chosen via simple randomization from the previously selected schools using stratified cluster sampling. All participants provided written informed consent (both in person and by their parents or legal guardians) to authorize access to the survey. Each student voluntarily took part in the survey, accessing the survey’s internet webpage at their school computer laboratory and used a unique identification number assigned randomly to ensure anonymity.

To maintain data integrity, participants were required to answer all questions, as the online system did not permit skipping without providing proper responses. However, data were considered to comprise missing values if there were logical errors or outliers. The raw data we used for analysis are publicly accessible on the KYRBS online site (https://www.cdc.go.kr/yhs/home.jsp (accessed on 9 June 2020)). Unfortunately, information on reasons for drop-out or non-participation were not available. Our study protocol for secondary analysis and interpretation was thoroughly reviewed and approved by the IRB of Samsung Changwon Hospital (IRB No. 2020-05-007).

### 2.2. Exclusion Criteria

Exclusion criteria were applied to participants with missing or implausible sleep records (e.g., instances where the specified sleep duration was unrealistically short, such as 30 min, or excessively long, such as 23 h). Specifically, of the initial 352,652 participants targeted for the study, 47,851 individuals were excluded due to inadequate or illogical sleep data, which were considered missing values.

### 2.3. Evaluation Indices

#### 2.3.1. Atopic Dermatitis (AD)

AD was diagnosed by the following question: “Have you ever been diagnosed with AD by a doctor at any point in your life or within the past 12 months”? The severity of disease, current symptoms, presence of treatment, and management modality were not assessed.

#### 2.3.2. Demographic and Socioeconomic Variables

Demographic and socioeconomic characteristics, including age, sex, school grade (middle school or high school), residential type, socioeconomic state (SES), and academic achievements, were assessed. Type of residence was assessed as either living with parents or living without parents (such as living with relatives, living independently such as in a dormitory, or in an orphanage). Degrees of SES and academic achievements were categorized as high (high or middle-high), middle (middle), or low (middle-low or low).

#### 2.3.3. Health-Related Behavioral Variables

The assessment of breakfast consumption frequency utilized the query, “During the previous 7 days, on how many days did you have breakfast”? Responses ranged from “never” to “7 days”. Participants reporting breakfast intake on fewer than two days weekly were designated as “breakfast skippers” according to the survey’s predefined criteria. We evaluated the frequency of vigorous exercise involving rapid breathing for over 20 min in the preceding seven days. Vigorous exercise was categorized as absent, occasional (1–3 days), and frequent (more than 4 days per week). Individuals were identified as current smokers if they indicated “more than 1 day” in response to the question: “How many days did you smoke even one cigarette during the last month before this survey”? Similarly, current drinkers were recognized as those who reported “more than 1 day” in response to the question: “How many days did you drink at least one shot glass of alcohol during the last month before this survey”?

#### 2.3.4. Emotional Variables including Suicidality

Psychological status was evaluated through four variables. Categorization of perceived healthiness included classifications of healthy, average, and unhealthy. The self-rated happiness scale determined the perceived level of happiness as happy, average, or unhappy. Perceived stress frequency was classified into often, sometimes, and rarely. Depression was assessed using the following question: “Within the last year, did you feel sad, blue, or depressed causing cessation of your usual activities almost every day for two weeks or more?” from the standpoint of KYRBS. The measure of suicidal ideation constituted a binary dependent variable (yes or no), and questions were structured as follows: “During the past 12 months, have you ever seriously considered committing suicide”? Assessments of recurring depressive mood and suicidal ideation were not conducted.

#### 2.3.5. Sleep

The primary independent variables in our study included weekday sleep duration, weekend sleep duration, and WCUS. In the KYRBSs, self-reported wake-up time and bedtime were determined on the basis of participants’ answers to the following questions, asked separately for weekdays and the weekend: (1) “What time did you usually go to bed and wake up on weekdays (school days) over the last week?” and (2) “What time did you usually go to bed and wake up on the weekend over the last week”? The responses were distinguished for weekdays and weekends as follows: (1) sleep time: ( ) o’clock ( ) minute AM/PM and (2) wake up time: ( ) o’clock ( ) minute AM/PM. The responses for sleep time on both weekdays and weekends were categorized as ≤9:00, 9:00–10:00, 10:00–11:00, 11:00 p.m.–12:00 a.m., 12:00–1:00, 1:00–2:00, and ≥2:00 a.m. Because wake-up time is influenced by school attendance, the responses for wake-up time were categorized as ≤5:00, 5–6:00, 6–7:00, 7:00–8:00, and ≥8:00 a.m. on weekdays and ≤7:00, 7:00–8:00, 8:00–9:00, 9:00–10:00, 10:00–11:00, and ≥11:00 a.m. on weekends. Based on the previous literature, we defined participants who go to sleep after 2 a.m. as night owls and those who wake up before 7 AM as early larks [10].

Sleep duration was also calculated separately for weekdays and weekends on the basis of the previous responses. Average sleep duration was calculated using the following weighted mean value [10]: (5 × weekday sleep duration + 2 × weekend sleep duration)/7, and categorized as ≤5, 5–6, 6–7, 7–8, 8–9, and ≥9 h. According to the National Sleep Foundation’s sleep time duration recommendations [33], sleep duration of 7–8 h per day was defined as the reference in this study. Furthermore, WCUS duration was calculated in hours in the present study by subtracting weekday sleep duration from weekend sleep duration, and then categorized into four groups: no catch-up, <1 h, 1–2 h, and ≥2 h. The four categories were used because the latter two groups occupied the first and second largest proportions of WCUS, while long WCUS was defined as sleeping at least 2 h longer on weekends than on weekdays [34].

Levels of sleep satisfaction were assessed by the degree of recovery from fatigue by sleep according to the following question: “How satisfied are you with your sleep during the last week”? and the responses were re-categorized as follows: enough (plenty and enough), a little, and not enough (not enough and never enough).

### 2.4. Statistical Analysis

We conducted all statistical analyses using complex sample procedures in the Statistical Package for the Social Sciences (SPSS) software program version 21.0 (IBM Corp., Armonk, NY, USA). As KYRBS data were collected through a representative, stratified, and clustered sampling method to be representative of the general population, data from the survey were weighed based on the sample design. The chi-square test for categorical variables and independent t-test for continuous variables were used to compare general characteristics between participants with AD and without AD. Following the selection of significant covariates by univariable logistic regression analysis, multivariate logistic regression analysis was carried out to determine which sleep-related factors independently contributed to the risk of suicidal ideation in AD according to the addition of variables: Model 1 was adjusted for sex and grade; Model 2 was adjusted for Model 1 variables + socioeconomic variables (residential type, socioeconomic state, and academic achievement) and health-related behavioral variables (breakfast skipping, smoking, drinking, and exercise); and Model 3 was adjusted for Model 2 variables + psychological variables (perceived healthiness, happiness, frequency of stress, and depression). The results are expressed with adjusted odds ratios (OR) and 95% confidence intervals (CI). Statistical significance was indicated in all tests by *p* < 0.05.

## 3. Results

### 3.1. Demographic Characteristics of Participants

During the five year study period, among a total of 352,652 targeted adolescents, 340,342 completed the survey with a total response rate of 96.5% (range 95.8% to 97.2%). Among these, 47,851 participants who did not provide logically consistent sleep records and whose data were considered as missing values were excluded, leaving a total of 71,434 adolescents with AD and 221,057 adolescents without AD. The overall prevalence rate of AD was 24.4%. Table 1 shows the general characteristics of participants and differences between participants with and without AD.

Participants with AD skipped breakfast less, exercised less frequently, drank alcohol less, and smoked less. Adolescents with AD self-reported unhealthier and unhappier perceptions, were stressed more often, had higher levels of depression, and had more frequent suicidal ideation and suicide attempts than participants without AD.

### 3.2. Sleep Parameters among Adolescents with and without Atopic Dermatitis

Adolescents with AD were significantly different from those without AD in terms of every sleep variable (Table 2).

Most importantly, sleep satisfaction was significantly poorer (not enough: 45.4% vs. 40.9%) in the AD group than in the non-AD group. Adolescents with AD slept less (average sleep duration 6.69 ± 1.47 vs. 7.63 ± 1.50 h and the proportion of less than 5 h of sleep duration was 8.1% vs. 7.2%, respectively) and were more likely to be night owls both on weekdays (20.8% vs. 18.6%) and during weekends (40.4% vs. 38.3%) compared with those without AD. However, adolescents with AD were more likely to be early larks on weekdays (46.2% vs. 44.8%) but less likely on weekends (6.3% vs. 6.7%) compared with those without AD. Although the absolute difference was small, mean weekday (5.94 ± 1.39 vs. 6.83 ± 1.42 h) and weekend (8.57 ± 2.88 vs. 9.63 ± 2.92 h) sleep durations were significantly shorter, while WCUS was significantly longer in adolescents with AD compared with those without AD (2.33 ± 1.79 vs. 1.95 ± 1.03 h). The proportion of long WCUS among adolescents with AD was 44.3% and it was 36.9% among adolescents without AD.

### 3.3. Associated Sleep Factors for Suicidal Ideation of Participants with Atopic Dermatitis

**Figure 1:**
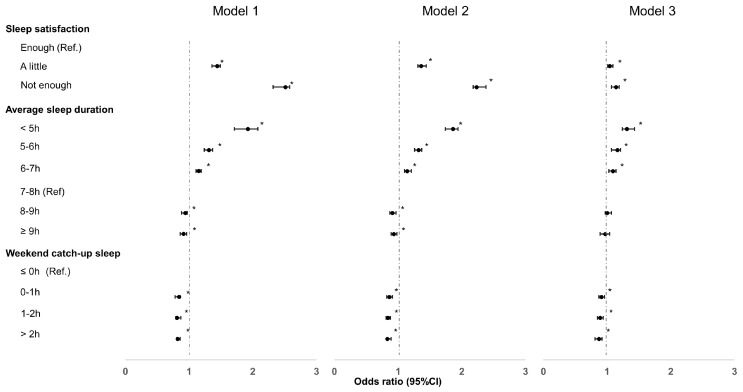
Adjusted odds ratio for suicidal ideation in adolescents with atopic dermatitis. NOTE. * *p* < 0.05: statistically significant. After the selection of significant covariates, univariate and multivariate logistic regression analyses were performed to identify factors associated with suicidal ideation in adolescents with AD. Model 1 was adjusted for sex and grade; Model 2 was adjusted for Model 1 variables + socioeconomic variables (residential type, socioeconomic state, and academic achievement) and health-related behavioral variables (breakfast skipping, smoking, alcohol drinking, and vigorous exercise); and Model 3 was adjusted for Model 2 variables + psychological variables (perceived status of health, perceived level of happiness, perceived frequency of stress, and experience of depressive symptoms).

Figure 1 illustrates the adjusted odds ratios (OR) for suicidal ideation based on various sleep parameters. Detailed information on the adjusted OR for suicidal ideation according to sleep parameters can be found in Table S1. The final results of adjusted OR after adjusting all variables at a time were revealed in those of Model 3.

After adjusting (Model 1), the OR of sleep dissatisfaction was 2.51 (95% CI, 2.32–2.58) times higher than for those with sleep satisfaction. In Models 2 and 3, the OR of sleep dissatisfaction was 2.23 (95% CI, 2.18–2.38) and 1.14 (95% CI, 1.07–1.19) times higher than for those with sleep satisfaction, respectively. In the average sleep duration, with 7–8 h as the reference, the OR of short sleep (≤5 h) was 1.92 (95% CI, 1.71–2.08) in Model 1, 1.86 (95% CI, 1.74–1.94) in Model 2, and 1.31 (95% CI, 1.24–1.43) in Model 3. Notably, long (≥2 h) WCUS was significantly associated with decreased suicidal ideation among adolescents with AD (OR: 0.82 (95% CI, 0.81–0.86) in Model 1, 0.83 (95% CI, 0.82–0.89) in Model 2, and 0.87 (95% CI, 0.81–0.92) in Model 3).

## 4. Discussion

The main findings of our study were that sleep dissatisfaction, short sleep, and AD itself were associated with increased risk of suicidal ideation. However, long WCUS was associated with decreased risk of suicidal ideation in Korean adolescents with AD. To the best of our knowledge, this is the first study to investigate the relationship between WCUS and suicidal ideation in adolescents with AD after adjusting for multiple potential confounding factors.

Our results indicate that short sleep duration is significantly associated with suicidal ideation in adolescents with AD. Although 8 to 10 h was considered appropriate for teenagers, and 7 to 9 h for young adults [33], polysomnography and actigraphy studies have found that children and adolescents with AD had significantly reduced sleep efficiency, longer sleep onset latency, daytime sleepiness, less non-rapid eye movement sleep, and difficulty falling back to sleep at night, are more restless in their sleep, and wake up more often [35,36]. However, in contrast with our study results and other reports that adults with AD have higher odds of shorter sleep duration (adjusted OR: 1.61 (95% CI, 1.16–2.25)) [37], it has also been reported that children and adolescents with AD have similar sleep duration, and bedtime and wake time as those without AD [35]. Although patients with AD were 44% more likely to exhibit suicidal ideation than patients without AD [23], the link between short sleep duration and suicidal ideation in adolescents with AD is complex and is yet to be fully understood.

There are several possible explanations for this association. For example, pruritus, a hallmark of AD, is thought to disrupt sleep by causing difficulty falling asleep and frequent nighttime awakenings because the itching often worsens at night, causing sequential mood changes in patients [7]. Severe and constant itching, the chronic and relapsing nature of AD, and a high comorbidity with other allergic diseases lead to stress and form a vicious cycle linked to an elevated risk of mental disorders including suicidality in adolescents with AD [5,38]. Scratching causes further damage to the skin barrier, stimulating skin inflammation, allowing for entry of irritants and pruritogens, and leading to an aberrant type 2 immune response, with increased immunoglobulin E (IgE) production, eosinophilia, mast cell activation, and overexpression of Th2 cytokines (interleukin [IL]-4, IL-5, IL-13) [38]. Filaggrin deficiency in AD also leads to an increase in cutaneous pH, which enhances the function of pruritogens that are upregulated in AD [7,39]. Elevated levels of neurotransmitters, including acetylcholine, histamine, and norepinephrine, in the skin of AD patients play a role in the sleep–wake cycle [4]. Melatonin, which is a major neurohormone that increases sleepiness, and decreases cutaneous inflammatory markers associated with AD, such as IL-4 and IgE, is decreased in AD patients during periods of exacerbation [4,36].

For adolescents, not only emotional stress but also academic stress should be considered as an associated factor for short sleep and related suicidality. Most Asian parents, especially those with high incomes, invest substantial resources in their children’s private education [40]. Likewise, many parents in Korea expect their offspring to achieve good results in education (outcomes such as admission to prestigious universities) in today’s competitive society. In Korea, most middle- and high-school classes start before 8:30 AM and after class most Korean adolescents attend private academic institutions or study at school late at night, even on weekends. Whatever the cause is, sleep deprivation is a stress-causing factor, with adolescents with short sleep having a higher risk of several mental disorders, including behavior disorders [41], substance abuse [41], depression [12,41,42], and suicidality [41].

Another notable finding of our study is that adolescents with AD are more likely to be night owls both on weekdays and during the weekends but are more often early larks on weekdays, though less on the weekend. These findings may be explained by the characteristics of AD itself and of education in Korea. In addition to increased nighttime awakenings and difficulty falling asleep, participants with active AD are more likely to report nightmares and early morning awakenings [6]. This may be related to longer WCUS in adolescents with AD than in those without AD due to the compensation of sleep debt accrued as a result of their sleep patterns, being both night owls and early larks on weekdays.

WCUS is a compensatory behavior in which people engage to cope with weekday sleep debt [12]. Because individual sleep needs vary, WCUS can reflect the degree of sleep insufficiency better than sleep duration and is an indicator of insufficient weekday sleep [10]. Although several studies in adults have shown that WCUS has a positive effect on human health [3,13,14], it is unclear whether WCUS has advantageous effects on emotional states because sleep hygiene guidelines recommend regularizing sleep time and wake-up time [12,33]. A recent study found that the risk for anxiety and depression was higher among adults who did not have WCUS than among those who did, [34] and long WCUS was associated with a decreased frequency of asthma and depression in Korean adolescents [10,12]. However, an association between WCUS and AD, particularly suicidal ideation among adolescents with AD, has not been identified. To the best of our knowledge, our results are the first to demonstrate that sufficient duration of WCUS to compensate for weekday sleep debt of ≥2 h may play a protective role against suicidal ideation among adolescents with AD. Nevertheless, adolescents with AD should have sufficient sleep time on weekdays, with WCUS also being an indicator of insufficient weekday sleep, while sleep debt leads to unfavorable health conditions and consequences [43].

This study has a number of limitations. First, the cross-sectional design meant that we could not establish causal relationships between associated sleep factors and suicidal ideation in adolescents with AD. Moreover, the sample size in this study was so large that statistical significance was easy to establish. We have presented many very low *p* values when comparing groups (see tables). However, the differences in actual numbers and percentages were tiny. Well-controlled prospective studies are necessary to confirm the results of the present study. Second, because only the lifelong diagnosis of AD was evaluated in this study, regardless of patients’ current states, we did not analyze the effects of management, treatment modality, or severity of AD due to the lack of such information in the survey. As many children suffering from AD go into remission before reaching adolescence, the definition of AD in this survey included both current AD and adolescents with a history of AD. This may have led to misclassifications of AD. Third, the use of a binary approach (yes/no) to assess the presence of suicidal ideation may not fully capture the complexity and severity of this phenomenon. This study relied on self-reported responses to a single question, and we recognize that suicidal ideation encompasses a spectrum of thoughts with varying levels of seriousness. Furthermore, we did not employ specific validated measurements designed to distinguish between different dimensions of suicidal thoughts, such as serious suicidal thoughts and passive suicidal ideation. This simplified approach may not fully capture the nuances associated with suicidal ideation.

Another limitation of this study was the self-reported nature of the data. In such a format, the credibility of responses regarding exact sleep time may be decreased. Studies using a more objective assessment of sleep, such as polysomnography and actigraphy, are required to resolve this limitation. In addition, despite various potential confounders that are adjusted for in this study, the survey did not assess other significant factors that have been associated with poor sleep quality, such as obstructive sleep apnea (OSA), snoring, nocturnal gastroesophageal reflux, internet or smartphone use time, and caffeine or drug use. In children and adolescents, a recent report has revealed that participants with AD have a higher risk of OSA than those without AD (hazard ratio, 1.86; 95% CI, 1.43–2.42) [44].

Despite these limitations, this study has several strengths and clinical implications that improve on the findings of previous reports on the mental health states of adolescents with sleep disorder among participants with AD. We used data from a nationwide, government-directed survey with a high response rate (96.5%). To the best of our knowledge, our study includes the largest number of participants among similar studies. A socioeconomically diverse sample with an equal proportion of middle- and high-school students (400 schools each annually) was included, and all analyses in this study were based on sample weights and adjusted for the complex sample design of the survey. Almost all participants had the same ethnic background, which minimized other possible confounding factors. These can allow for generalization of the results in Korea.

Most of all, this research identified associated sleep factors for suicidal ideation in adolescents with AD, and there has been no study on the relationship between WCUS and AD. The public health implications of our findings can be applied at both the educational and practical levels. From an educational perspective, strategic interventions to reduce academic stress on Korean adolescents should be seriously considered, such as a later start of classes. Healthcare professionals, including pediatricians and dermatologists, need to be more aware of the risk of mental health problems and suicidality in adolescents with AD, particularly amongst those with sleep problems. When treating adolescents with AD, screening procedures for associated sleep factors related to suicidality, including short sleep duration and sleep dissatisfaction, should be emphasized.

In conclusion, though the conclusions of this study should be considered continuously, our findings suggest that many adolescents with AD in Korea sleep less and have greater sleep dissatisfaction and longer WCUS than those without AD. Long WCUS, an indicator of the compensatory effect of insufficient weekday sleep, is associated with a decreased risk of suicidal ideation in Korean adolescents with AD. It is critical for adolescents with AD to obtain sufficient sleep to reduce suicidal ideation.

## Figures and Tables

**Table 1:** General characteristics and emotional states of study participants.

	Presence of Atopic Dermatitis		
	No (n = 221,057)	Yes (n = 71,434)	*p*-Value
Sex			<0.001
Female	104,818 (46.28)	40,751 (55.87)	
Male	116,239 (53.72)	30,683 (44.13)	
Grade			<0.001
Middle school	108,055 (45.89)	34,027 (44.89)	
High school	113,002 (54.11)	37,407 (55.11)	
Subjective economic state			<0.001
High	78,817 (36.11)	24,558 (34.76)	
Middle	105,294 (47.39)	34,087 (47.51)	
Low	36,946 (16.50)	12,789 (17.73)	
Academic achievement			<0.001
High	82,770 (37.29)	28,624 (39.76)	
Middle	62,925 (28.62)	19,907 (28.04)	
Low	75,362 (34.09)	22,903 (32.21)	
Residential type			0.980
With parents	210,825 (95.92)	68,120 (95.92)	
Without parents	10,232 (4.08)	3314 (4.08)	
Breakfast consumption			<0.001
<2 times/week	46,089 (20.78)	14,300 (20.04)	
≥2 times/week	174,968 (79.22)	57,134 (79.96)	
Vigorous exercise >20 min/week			<0.001
No	50,957 (23.37)	17,636 (25.13)	
Occasional	119,830 (54.44)	39,183 (55.10)	
Frequent	50,270 (22.19)	14,615 (19.78)	
Current drinking			0.038
No	186,615 (84.01)	60,550 (84.39)	
Yes	34,442 (15.99)	10,884 (15.61)	
Current smoking			<0.001
No	204,356 (92.21)	67,027 (93.63)	
Yes	16,701 (7.79)	4407 (6.37)	
Subjective healthiness			<0.001
Healthy	161,194 (72.85)	47,748 (66.68)	
Average	47,519 (21.53)	18,000 (25.30)	
Unhealthy	12,344 (5.63)	5686 (8.02)	
Subjective happiness			<0.001
Happy	115,977 (64.08)	35,784 (61.78)	
Average	48,884 (27.36)	16,199 (28.29)	
Unhappy	15,218 (8.56)	5663 (9.93)	
Perceived stress			<0.001
Often	80,806 (36.60)	29,582 (41.30)	
Sometimes	95,178 (43.23)	30,115 (42.44)	
Rare	45,073 (20.16)	11,737 (16.26)	
Depression			<0.001
No	165,975 (74.96)	50,836 (71.12)	
Yes	55,082 (25.04)	20,598 (28.88)	
Suicidal ideation			<0.001
No	194,272 (87.81)	60,946 (85.43)	
Yes	26,785 (12.19)	10,488 (14.57)	
Suicidal attempts			<0.001
No	215,537 (97.52)	69,353 (97.10)	
Yes	5520 (2.48)	2081 (2.90)	

NOTE. Survey data were weighted for statistical representation of the general population based on the sample design. The chi-square test was applied to determine significant differences between categorical data, and the independent *t*-test was used for continuous variables.

**Table 2:** Comparisons of sleep-related variables between participants with and without atopic dermatitis.

	Presence of Atopic Dermatitis		
	No (n = 221,057)	Yes (n = 71,434)	*p*-Value
Sleep satisfaction			<0.001
Enough	59,576 (26.37)	16,637 (23.02)	
A little	72,242 (32.79)	22,532 (31.56)	
Not enough	89,239 (40.85)	32,265 (45.43)	
Weekday			
Sleep time			<0.001
<21:00	330 (0.13)	84 (0.11)	
21:00–22:00	2964 (1.15)	686 (0.82)	
22:00–23:00	17,478 (6.84)	4610 (5.65)	
23:00–24:00	50,707 (21.50)	14,807 (19.55)	
24:00–1:00	56,912 (26.03)	18,340 (25.64)	
1:00–2:00	54,537 (25.75)	18,987 (27.41)	
≥2:00	38,129 (18.59)	13,920 (20.82)	
Wake-up time			<0.001
<5:00	732 (0.33)	236 (0.30)	
5:00–6:00	9319 (3.84)	3238 (4.23)	
6:00–7:00	90,022 (40.59)	29,921 (41.63)	
7:00–8:00	112,321 (51.00)	35,478 (49.90)	
≥8:00	8663 (4.24)	2561 (3.94)	
Sleep duration, h	6.83 ± 1.42	5.94 ± 1.39	<0.001
Weekend day			
Sleep time			<0.001
<21:00	452 (0.19)	135 (0.17)	
21:00–22:00	1952 (0.76)	487 (0.59)	
22:00–23:00	10,464 (4.23)	2846 (3.61)	
23:00–24:00	31,480 (13.26)	9263 (12.11)	
24:00–01:00	49,041 (21.85)	15,290 (21.16)	
01:00–02:00	46,154 (21.42)	15,446 (21.97)	
≥02:00	81,514 (38.29)	27,967 (40.40)	
Wake-up time			<0.001
<7:00	15,559 (6.72)	4555 (6.27)	
7:00–8:00	25,992 (11.48)	8327 (11.38)	
8:00–9:00	48,241 (21.70)	15,440 (21.67)	
9:00–10:00	47,070 (21.52)	15,343 (21.67)	
10:00–11:00	40,813 (18.73)	13,192 (18.60)	
≥11:00	43,382 (19.85)	14,577 (20.41)	
Sleep duration, h	9.63 ± 2.92	8.57 ± 2.88	<0.001
Average sleep duration, h	7.63 ± 1.50	6.69 ± 1.47	<0.001
Average sleep duration			<0.001
<5 h	14,853 (7.15)	5468 (8.09)	
5–6 h	36,697 (17.52)	13,259 (19.39)	
6–7 h	55,239 (25.76)	18,701 (26.70)	
7–8 h	56,754 (25.25)	17,614 (24.19)	
8–9 h	38,396 (16.30)	11,097 (14.68)	
≥9 h	19,118 (8.02)	5295 (6.95)	
Weekend catch-up sleep	1.95 ± 1.03	2.33 ± 1.79	<0.001
≤0 h	35,765 (15.80)	10,459 (14.47)	
0–1	52,461 (24.88)	13,357 (18.64)	
1–2	49,017 (22.45)	15,984 (22.59)	
>2	83,814 (36.87)	31,634 (44.29)	

Note: Average sleep duration: (weekday sleep duration × 5 + weekend day sleep duration × 2)/7, Weekend catch-up sleep: weekend day sleep duration minus weekday sleep duration.

## Data Availability

Availability of data and material Raw data are available on the official website of KYRBS, https://www.cdc.go.kr/yhs/home.jsp (accessed on 9 June 2020).

## Supplementary Materials

The following supporting information can be downloaded at: https://www.mdpi.com/article/10.3390/jcm12247716/s1, Table S1. Adjusted odds ratio for suicidal ideation in adolescents with atopic dermatitis.
