# Supplementary material for: Association of Sleep Duration and Weekend Catch-Up Sleep with Suicidal Ideation among Adolescents with Atopic Dermatitis
Source: J Clin Med. 2023 Dec 15;12(24):7716. doi: 10.3390/jcm12247716 (PMC10743835; doi:10.3390/jcm12247716)
Supplement: Supplementary file 1 [file jcm-12-07716-s001.zip › jcm-2744609-supplementary.pdf]

Supplementary materials

Table S1. Adjusted odds ratio for suicidal ideation in adolescents with atopic dermatitis.

	Suicidal ideation					
	Model 1 OR (95% CI)	p-value	Model 2 OR (95% CI)	p-value	Model 3 OR (95%CI)	p-value
**Sleep satisfaction**						
Enough	Reference		Reference		Reference	
A little	1.44 (1.36-1.49)	<0.001	1.36 (1.31-1.44)	<0.001	1.04 (1.01-1.09)	<0.001
Not enough	2.51 (2.32-2.58)	<0.001	2.23 (2.18-2.38)	<0.001	1.14 (1.07-1.19)	<0.001
**Average sleep duration**						
<5hr	1.92 (1.71-2.08)	<0.001	1.86 (1.74-1.94)	<0.001	1.31 (1.24-1.43)	<0.001
5-6hr	1.31 (1.24-1.37)	<0.001	1.32 (1.26-1.37)	<0.001	1.16 (1.07-1.21)	<0.001
6-7hr	1.15 (1.11-1.19)	<0.001	1.14 (1.10-1.21)	<0.001	1.09 (1.03-1.14)	0.003
7-8hr	Reference		Reference		Reference	
8-9hr	0.94 (0.88-0.97)	<0.001	0.91 (0.87-0.97)	<0.001	1.00 (0.97-1.07)	0.736
≥9hr	0.91 (0.86-0.96)	0.003	0.93 (0.89-0.98)	0.015	0.97 (0.89-1.04)	0.917
**Weekend catch-up sleep**						
≤0hr	Reference		Reference		Reference	
0-1hr	0.84 (0.78-0.86)	<0.001	0.86 (0.82-0.91)	<0.001	0.91 (0.87-0.96)	<0.001
1-2hr	0.81 (0.79-0.87)	<0.001	0.84 (0.81-0.88)	<0.001	0.89 (0.85-0.94)	<0.001
>2hr	0.82 (0.81-0.86)	<0.001	0.83 (0.82-0.89)	<0.001	0.87 (0.81-0.92)	<0.001

Abbreviations: OR, odds ratio; CI, confidence interval

NOTE. After the selection of significant covariates, univariate and multivariate logistic regression analyses were performed to identify factors associated with suicidal ideation in adolescents with AD. Model 1 was adjusted for sex and grade; Model 2 was adjusted for Model 1 variables + socioeconomic variables (residential type, socioeconomic state, and academic achievement) and health-related behavioral variables (breakfast skipping, smoking, alcohol drinking, and vigorous exercise); and Model 3 was adjusted for Model 2 variables + psychological variables (perceived status of health, perceived level of happiness, perceived frequency of stress, and experience of depressive symptoms).
